# Engaging the Next Generation: Designing an Experiential Intensive Care Unit Workshop on Neurologic Emergencies for Medical Students From Diverse Backgrounds

**DOI:** 10.1111/tct.70407

**Published:** 2026-03-31

**Authors:** Marie Guinat, Danai‐Georgia Bucher, Yoann Salnave, Maja Arnold, Laura Westermann, Coline Glauser, Nawfel Ben‐Hamouda

**Affiliations:** ^1^ Department of Intensive Care Medicine Lausanne University Hospital (CHUV) Lausanne Switzerland; ^2^ Medical Education Unit of the School of Medicine, Faculty of Biology and Medicine (FBM) University of Lausanne Lausanne Switzerland; ^3^ Faculty of Biology and Medicine (FBM), School of Medicine University of Lausanne Lausanne Switzerland

**Keywords:** experiential learning, gamification, neurologic emergencies, peer teaching, student‐led initiative

## Abstract

**Background:**

Undergraduate medical students often feel unprepared to manage unstable patients, especially in emergency and intensive care settings. Inadequate exposure to real clinical learning tasks without hands‐on experience may contribute to these feelings of unpreparedness. Traditional lectures might not sufficiently support the development of medical skills in this area. This article describes the design and satisfaction‐based evaluation of a 1‐h innovative workshop for teaching undergraduate medical students how to manage unstable neurologic patients.

**Approach:**

In March 2025, the *Swiss Medical Students' Convention* organised by the *Swiss Medical Students' Association* took place in Lausanne, Switzerland, with the goal of improving students' readiness to manage unstable patients in an acute care environment. The workshop included students from diverse Swiss universities and training backgrounds, which called for a flexible and inclusive approach. It employed experiential learning strategies and gamification principles to engage students in three structured stations, focusing on neurologic assessment, identifying red flags in comatose patients and preventing secondary cerebral damage.

**Evaluation:**

After the workshop, students completed a questionnaire with closed and open‐ended questions. Quantitative and qualitative data were analysed using descriptive statistics.

Data indicated a high level of satisfaction among participants regarding the structure, content and interactivity of the workshop. Medical students appreciated the peer‐based, mixed‐level learning approach, which promoted collaboration and critical thinking.

**Implications:**

This initiative demonstrates how short, hands‐on, student‐centred workshops can enhance medical students' engagement for critical care management. Future research should include preassessment and postassessment to measure its impact on clinical reasoning and knowledge growth.

## 1 Background

Critical care training is a demanding process that requires professionals to acquire and sustain the knowledge, skills and attitudes necessary for providing optimal patient care [1]. However, medical students do not feel adequately prepared to manage unstable patients, reporting low self‐confidence in emergency care and requiring close supervision [2]. These feelings of insufficient preparedness can result from limited exposure to a variety of critically ill patients during undergraduate training [3]. Undergraduate curricula should include more hands‐on experiences to help students develop reflective and clinical reasoning skills to manage critically ill patients [4]. Teaching these abilities is challenging because patient safety restrictions limit students' involvement in patient care. Therefore, the traditional classroom lecture remains one of the most common teaching methods for intensive care, but educators should prioritise developing teaching approaches to address the educational challenges of the intensive care unit (ICU) environment [5].

Workshops, as a collaborative teaching method, represent an innovative alternative to traditional lectures. They enable the integration of adult learning principles and encourage learners to reflect on their experience [6]. This approach may support the development of clinical competence by allowing learners to apply knowledge, skills and attitudes in an integrated way during complex acute care situations [7]. Recommendations on learning emphasise that learning should be active, contextual and collaborative. There is a growing emphasis on learner‐centered and experiential learning approaches alongside the integration of innovation and technology in education. In this regard, the Lewinian experiential learning model emphasized the importance of concrete experiences and immediate feedback [8]. The four steps of Kolb's learning cycle (concrete experience, reflective observation, abstract conceptualisation and active experimentation) guide educational designers in engaging learners in new teaching experiences that challenge existing cognitive schemata through active reflection on action.

These principles guide the design and implementation of experiential and interactive workshops, where learners can actively engage with the simulated environment, making the learning experience more memorable than simply watching or listening to a traditional lecture. According to Mayer's theory, people learn better when words and images are combined to explain information, rather than using only one or the other [9]. Additionally, incorporating gamification principles into workshop design can boost learners' motivation and performance [10]. Workshops that actively involve students in authentic situations are a teaching strategy that may align well with the goals of intensive care education [11].

This article focuses on a student‐led initiative that promoted teaching in the ICU. The 38th edition of the *Swiss Medical Students' Convention* (SMSC), organised by the *Swiss Medical Students' Association*, took place in Lausanne in 2025. The SMSC consisted of a congress that brought together medical students from across Switzerland to learn about topics outside traditional lecture. This year's theme ‘Intensive Care Medicine – Critical Care, Vital Solutions’ brought together students and professionals from all over Switzerland to explore the complexities of critical care. Professionals from emergency and intensive care departments proposed learning activities to teach patient management in acute healthcare settings. The organisational challenge was the large number of students, over 350, from different universities with varying levels of training. Several workshops were organised to provide exposure to various subtopics in ICU, such as basic life support or neurological disorders.

This article describes the design and evaluation of a 1‐h workshop aimed at teaching medical students of different levels of training about the management of neurologic disease in the ICU.

## 2 Approach

### 2.1 Participants

The participants were medical students from five different Swiss universities and one international student from Austria, with different levels of training, from the first year (Bachelor's one) to the last year of training (Master's three). All participants registered for the workshops online on a first‐come, first‐served basis 3 weeks before the convention.

### 2.2 Description of the Design of the Workshop

A 1‐h workshop featuring various stations was developed following the ADDIE model, a flexible framework adaptable to various teaching formats, with an initial need analysis, specifically relevant for the ICU, where high‐acuity situations require rapid management and alignment between learning objectives and educational activities.

#### 2.2.1 Analysis

Mixed student subgroups were designed based on an initial analysis of the student population, taking into account differences in educational background, university curricula and level of training. Despite this heterogeneity, all students shared a common foundation in pathophysiology and basic sciences, usually introduced in the first year of medical training, which serves as the baseline for the workshop.

#### 2.2.2 Design

Based on the analysis of this educational context, two academic experts in ICU designed the learning objectives of the workshop:

- Assess a comatose patient,
- Recognise the Red flag in the neuro ICU and
- Understand and identify the basic management of acute neurologic disease (haemorrhage, traumatic disease …), more specifically, the secondary cerebral aggression of systemic origin

#### 2.2.3 Development

Anchored in these learning objectives, three stations were developed to align educational activities with the desired outcomes. The learning activities were conceived using a whole‐task approach, to support the integration of the knowledge, skills and attitudes required to manage clinical situations. Activities were structured to mirror real‐life professional tasks students may encounter, facilitating the transfer of acquired competencies to clinical practice. The different stations of the workshop are described in Table 1:

- Station 1: ‘*Short puzzle game on neurologic assessment*’
- Station 2: ‘*Online Quizz (* wooclap.com *): If my patient is comatose… What should I be aware of*?’
- Station 3: ‘Room of errors: Find the errors on the secondary cerebral aggression of systemic origin’

**TABLE 1:** Description of the different phases of the workshop.

Allotted time and learning objectives	Description of the activity	Learning principles used in the design of the activity	Expected positive effects on learning	Role of the facilitator
10 min	**Introduction of the workshop**			
Present the outline of the workshop	Facilitators welcome participants to the workshop and introduce themselves. They explain learning objectives and a session agenda. They form small groups of learners with heterogeneous backgrounds.		‐ Create a safe learning environment. ‐ Foster peer feedback and feedback seeking. ‐Promote collaboration through learning.	‐ Set the rule of the game: no stress, no judgement, no shame. ‐ Promote effective learning and team functioning. ‐ Sustain students' motivation.
15 min	**Station 1: ‘*Online Quizz: If my patient is comatose… What should I be aware of*?’**			
Recognise the red flag while assessing and managing a comatose patient. Describe the physiopathology and clinical presentation of common neurocritical patient presentations.	With an interactive questioning platform (named Wooclap), students were exposed to a case‐based situation through a narrative oral presentation using PowerPoint to reflect on red flags for different neuro ICU management and what they should be aware of while managing a comatose patient.	*Mayer's multimedia learning theory* The 12 principles of optimal multimedia design were used to design the interactive presentation.	‐ Offer better integration of new information with prior knowledge. ‐ Foster deep learning.	‐ Stimulate and foster reflection through real case based discussions. ‐ Support students in hypothesis generation.
15 min	**Station 2: ‘*Room of errors: Find the errors on the secondary cerebral aggression of systemic origin*’**			
Understand and identify the fundamental management of acute neurologic diseases (such as haemorrhage, traumatic injury, etc.), with an emphasis on recognising and reducing secondary brain injury caused by systemic factors. Apply pathophysiological knowledge to recommend appropriate corrective actions to limit secondary cerebral insults.	Students engaged in a scenario with a high‐fidelity mannequin set in an ICU environment, where the patient was admitted after a head injury from a scooter accident. Various errors were embedded in this specific ICU setting. Students, as a group, were expected to identify these errors, explain how they could notice them in the simulated environment and suggest ways to correct them.	*Experiential learning theory with the four steps of Kolb's cycle* **Concrete experience**: Students used the identified errors to reflect on their effect and physiopathological impact on patient outcomes. **Reflective observation:** Students analysed how they were able to identify the errors. **Abstract conceptualisation:** Students made general principles or rules and developed a shared mental model to understand and anticipate these errors with other patients (prehospital context, anaesthesiology …). **Active experimentation:** Students proposed and applied corrective strategies to correct the errors and prevent recurrence.	‐ Promote reflection on actions while resolving concrete problems. ‐ Increase competence levels through experiences. ‐ Transfer and apply theoretical knowledge in authentic experiences.	‐ Prepare learners for deeper clinical reasoning discussions during debriefing.
15 min	**Station 3: ‘*Short puzzle game on neurologic assessment*’**			
Assess a comatose patient using the Glasgow scale. Match neurological emergencies with appropriate treatments using pathophysiological reasoning.	In this interactive station, students began by analysing six paper‐based clinical scenarios involving comatose patients with altered Glasgow Coma Scale (GCS) scores. They were asked to determine each patient's GCS score based on the clinical description. Upon correctly calculating the scores, students received an envelope containing a matching puzzle activity. Then, in the next step students were expected to match neurological conditions with their appropriate antidotes or treatments and explain the mechanisms using pocket cards, following puzzle principles. By arranging the pocket cards in the correct order, a magic question appeared: *When evaluating a patient with a neurological disorder, what standardized approach could be used for a comprehensive and systematic assessment?* After providing the correct answer, which was ‘ABCDE approach,’ there was an open discussion about the Glasgow assessment and the ABCDE approach with the students and one of the teachers.	*Self‐determination theory* **Autonomy**: Participation was voluntary as students chose to register for the workshop during the student‐led congress. **Competence**: Learning tasks were challenging for a short time, but goals were achievable through collaboration within the subgroup of students. **Relatedness**: Social connection and interaction might be a source for finding the key to the play, depending on students' diverse expertise and interconnectedness.	‐ Promote real‐life medical decision‐making complexities ‐ Engage the learners actively in learning, enhancing motivation	‐ Support inquiry. ‐ Ensure correct understanding of clinical assessment and clinical problem. ‐ Adopt ‘over‐the‐shoulder’ feedback, guiding inquiry, managing time. ‐ Prompt discussion when needed, with respect to the collaborative dynamics of the group.
5 min	Finally, at the end of the workshop, participants received pocket guidelines with simplified TIPS for ICU neurologic care, designed to facilitate better knowledge retrieval, understanding of the learning experience and application in clinical practice.			

#### 2.2.4 Implementation

During the implementation, the expected number of students per workshop session was between 10 and 15. Three mixed student subgroups were deliberately assembled with diverse levels of training to promote peer learning and collaborative practice, ensuring each group included final‐year students. Each subgroup consisted of up to five students, who were expected to take turns at each 15‐min station. Final‐year students were briefed to support younger students through peer teaching, explicitly articulating their clinical reasoning and facilitating group reflection. This role for senior students was intended to provide educational benefits, as assuming a teaching role allowed students to consolidate and test their competence. During the workshop, students were guided by two academic experts in ICU, one with specific expertise in neurological complications (Anonymous) and another with expertise in medical education (Anonymous). The experts provided just‐in‐time, ‘over‐the‐shoulder’ guidance, offering targeted feedback and facilitating reflection without interrupting students' autonomy. Finally, at the end of the workshop, students received pocket guides with tips for ICU neurologic care to improve knowledge retention.

*Final‐year students were briefed to support younger students through peer teaching, explicitly articulating their clinical reasoning and facilitating group reflection*.

#### 2.2.5 Evaluation

Students' feedback was used to assess whether the workshop meets the educational objectives as described below.

### 2.3 Evaluation

A postworkshop questionnaire was elaborated to assess students' satisfaction with the workshop. Active learning strategies, authenticity of the scenario and structured reflection were central components of the workshop. Accordingly, the questionnaire items were designed to align specifically with these outcomes.

Forty medical students took part in the workshop in March 2025. Most students were women (23/40, 57%) aged between 20 and 31 years. The majority of participants were from Lausanne University (16/40, 40%). Table 2 provides details on the number of students involved in the workshop.

**TABLE 2:** Demographic table of participants involved in the workshop.

Number of medical students involved in the course (*n* = 40)	*n* (%)
Gender	
Female	23 (57.5)
Male	17 (42.5)
Age	
20–25 years	37 (92.5)
26–30 years	0 (0)
> 30 years	3 (7.5)
Level of training	
Bachelor	26 (65.0)
Master	14 (35.0)
University of training	
Lausanne	17 (42.5)
Fribourg	8 (20.0)
Zurich	7 (17.5)
Bern	4 (10.0)
Bale	4 (10.0)

Quantitative and qualitative data were gathered from participants after they attended the workshop. Participants completed a questionnaire that included a closed‐item questionnaire and open‐ended questions. They responded to nine closed questions using a 5‐point Likert scale ranging from 1 (strongly disagree) to 5 (strongly agree), with a neutral midpoint.

### 2.4 Ethics Statement

The study was approved by the Ethics Committee of the Canton de Vaud, Switzerland (CER‐VD: Req‐2025‐00954**).**

## 3 Results

The questionnaire response rate was 90%. Four students involved in organizing the 2‐day congress did not evaluate the workshop.

### 3.1 Responses to Closed‐Ended Questions

**FIGURE 1:**
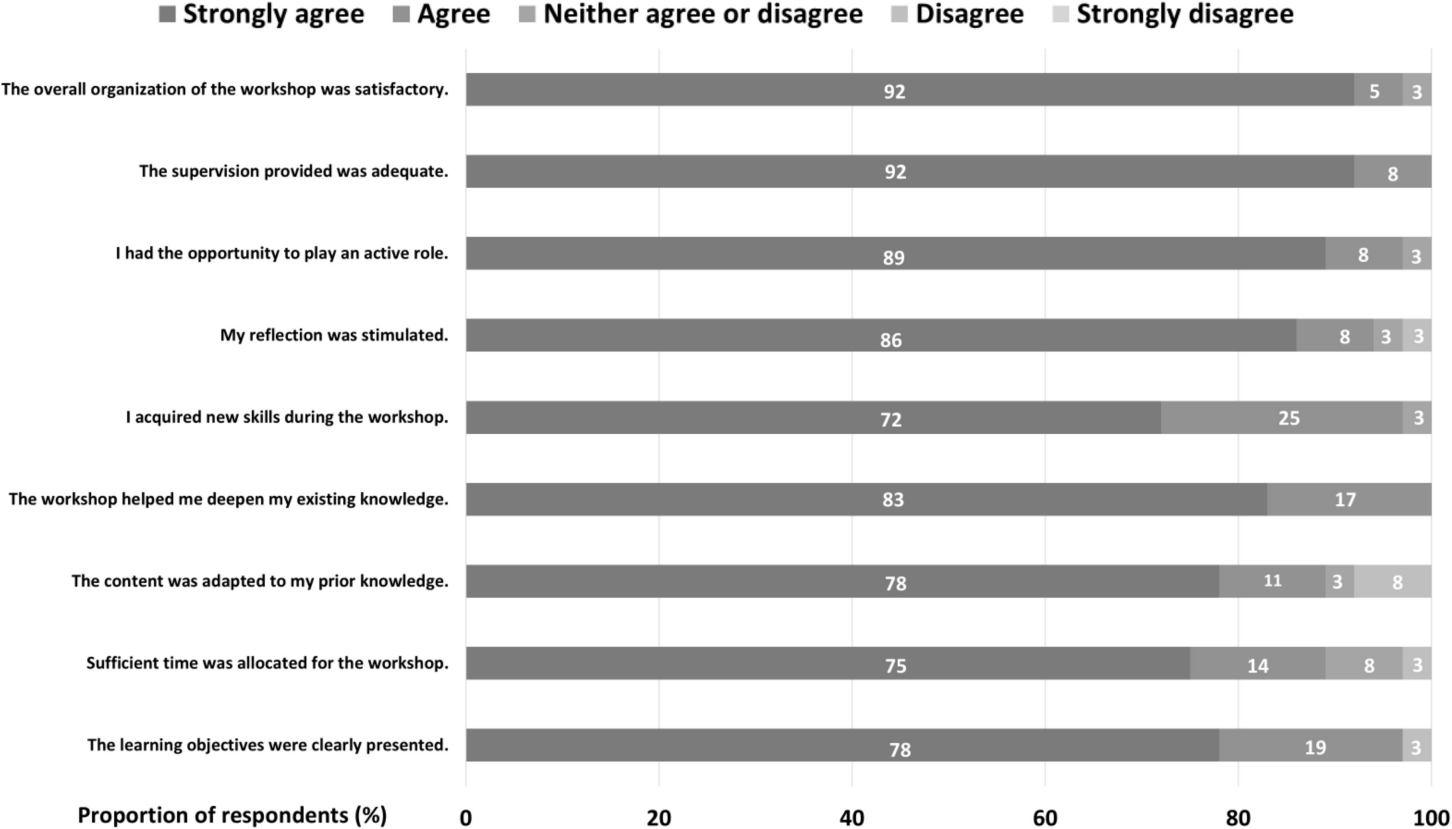
Quantitative evaluation of the workshop.

Most participants either strongly agreed or agreed that the overall organisation of the workshop was satisfactory. Figure 1 provides more details on the responses to each question.

### 3.2 Responses to Open‐Ended Questions

Table 3 presents examples of the quotes collected after the workshop. Participants appreciated the interactive design of the workshop that allowed them to be active and apply their knowledge concretely. They valued the reflective environment with peer collaboration.

**TABLE 3:** Selected quotes from the participants.

Illustrative quotes on participants' appreciation of the workshop
**What did you appreciate in the workshop?**
‘It was really great to be able to actually do things rather than just listen.’ ‘Active and participatory reflection.’ ‘I was able to reflect and apply my knowledge.’ ‘A dynamic workshop with helpful reminders of physiology.’ ‘We retained the essentials, applied our knowledge, and made links with pharmacology. This approach should definitely be used in teaching.’ ‘I appreciated the division into different stations and the opportunity to do something practical’ ‘Three activities in one, with a good overall approach.’ ‘Interactive presentation in a ‘game‐based’ format made it more enjoyable.’ ‘The playful aspect helped us retain the key points.’ ‘Practical, concrete situations. Several different activities.’ ‘A practical workshop with little theory and a strong game‐based component.’ ‘Interesting! My prior knowledge was limited, but I learned a lot.’ ‘It was great to work with students from different academic years.’ ‘The practical situation was very good, with good interaction with physicians.’ ‘Everything was appreciated. It was excellent to let us think as a group and then guide us. Giving space for reflection was really valuable’
**What could be improved in the workshop?**
‘A little bit more time’

*Participants appreciated the interactive design of the workshop that allowed them to be active and apply their knowledge concretely*.

## 4 Implications

This article describes the design of a workshop aimed at teaching medical students from various Swiss universities how to assess unconscious patients in ICU settings as frontline medical practitioners. This innovative workshop was grounded in constructivist learning theory and aligned with Kolb's experiential learning cycle. It combined self‐directed strategies and peer‐supported learning within realistic ICU scenarios. The satisfaction evaluation showed a high level of student satisfaction, suggesting that this format is well‐suited to medical students with diverse backgrounds in critical care education.

These findings are consistent with the literature, which shows that gamification enhances students' engagement in the learning process and promotes higher‐level thinking [12]. Gamification, increasingly described in the ICU context, relies on elements such as competition, role‐playing and the integration of games into the clinical environment. Following these principles, this workshop engaged learners in concrete, meaningful clinical scenarios grounded in real‐life situations. It fosters a rich learning environment where students can actively participate in solving problems and connect knowledge to practice [13]. This gamification approach was also implemented using a room‐of‐error, a method for identifying patient safety hazards [14]. In this setting, student interaction supports rapid identification and correction of realistic problems students might encounter in clinical training, thereby improving situational awareness [15]. During the described workshop, interaction among students was facilitated by creating mixed‐level subgroups that included students from diverse backgrounds. More specifically, peer feedback helps fill knowledge gaps, foster mutual support and improve shared expertise [15]. Additionally, two ICU experts facilitated debriefings. Together, peer and expert feedback enhanced clinical reasoning and contributed to achieving the workshop's learning outcomes [8].

*This workshop engaged learners in concrete, meaningful clinical scenarios grounded in real‐life situations*.

Future improvements should include the analysis of pre‐ and postworkshop performance, as well as the assessment of long‐term skill retention to better determine whether workshops of this type can effectively bridge the gap between theoretical knowledge and practical ICU skills.

## 5 Limitations

This study has several limitations. The student population may limit the generalizability of our findings. Second, the workshop assessment focused solely on students' satisfaction rather than competency outcomes. Third, specific components of the instructional design that contributed to learning outcomes were not examined. Finally, voluntary participation in the workshop may have introduced selection bias.

## 6 Conclusion

An experiential, gamified and peer‐supported workshop is feasible and effectively engages medical students from diverse backgrounds in critical care education.

## Author Contributions

**Marie Guinat:** conceptualization, formal analysis, investigation, methodology, project administration, resources, visualization, supervision, writing – original draft preparation, writing – review and editing. **Danai‐Georgia Bucher:** conceptualization, data curation, writing – review and editing. **Yoann Salnave:** conceptualization, data curation, writing – review and editing. **Maja Arnold:** conceptualization, data curation, writing – review and editing. **Laura Westermann:** conceptualization, data curation, writing – review and editing. **Coline Glauser:** conceptualization, data curation, writing – review and editing. **Nawfel Ben‐Hamouda:** conceptualization, formal analysis, investigation, methodology, project administration, resources, visualization, supervision, writing – original draft preparation, writing – review and editing.

## Funding

The authors received no specific funding for this work.

## Ethics Statement

The study was approved by the Ethics Committee of the Canton de Vaud, Switzerland (CER‐VD: Req‐2025‐00954**).** The questionnaires reported were anonymous and conducted voluntarily. Participants were informed that completing and returning the online survey was taken as informed consent. Thus, there was no need for a formal collection of informed consent. Participants were informed that completing and returning the online survey constitutes informed consent for publication. All participants were allowed to ask questions and provided contact information for the research team in case they had further concerns about the study.

## Conflicts of Interest

The authors declare no conflicts of interest.

## Data Availability

The data that support the findings of this study are available from the corresponding author upon reasonable request.
